# Short‐term effects of exposure to workplace bullying on objective sleep: an actigraphy diary study

**DOI:** 10.1111/jsr.14412

**Published:** 2024-11-27

**Authors:** Alfredo Rodríguez‐Muñoz, Mirko Antino, Alejandro Díaz‐Guerra, Ana Isabel Sanz‐Vergel, Arnold B. Bakker

**Affiliations:** ^1^ Universidad Complutense de Madrid Madrid Spain; ^2^ Norwich Business School, University of East Anglia Norwich UK; ^3^ Erasmus University Rotterdam Rotterdam The Netherlands

**Keywords:** anxiety, diary, objective sleep, workplace bullying

## Abstract

Exposure to bullying behaviours has been associated with a variety of negative health outcomes, such as sleep complaints. However, the current state of the knowledge is limited regarding the association with objective sleep. The present study investigated the short‐term effects of workplace bullying on objective sleep patterns using an actigraphy diary approach. Participants (*N* = 55) wore actigraphy devices for 10 days to measure sleep parameters such as duration, wake‐after‐sleep onset (WASO), and the number of awakenings. Multilevel analyses showed that exposure to workplace bullying was directly associated with the three parameters of sleep disturbances, with higher levels of bullying linked to poorer sleep outcomes. Anxiety was also found to mediate this relationship. Specifically, anxiety mediated the association between bullying and WASO and sleep duration. The study contributes valuable insights into the detrimental impact of workplace bullying on objective sleep quality, highlighting the importance of addressing psychosocial stressors in the workplace to promote healthy sleep patterns.

## 1 INTRODUCTION

In recent years, interest in sleep within organisational psychology has grown, especially regarding its link to work‐related stressors. Workplace bullying is considered one of the most detrimental psychosocial stressors (Samnani & Singh, 2012), and refers to a form of mistreatment in which one or more employees are repeatedly exposed to negative social acts of a primarily psychological nature (Einarsen et al., 2020). A key aspect of bullying is the persistence of these behaviours over time. Most definitions include a minimum duration of exposure to negative acts of at least 6 months (Einarsen et al., 2020). However, this timeframe has been criticised as arbitrary (Nielsen et al., 2011), as bullying likely exists on a continuum, from occasional exposure to severe victimisation (Notelaers & Einarsen, 2013), and should be studied as a complex phenomenon rather than a binary. Consequently, researchers have recently begun to investigate short‐term effects, conducting studies over periods shorter than 6 months, including weekly (Tuckey & Neall, 2014) and daily observation periods (Rodríguez‐Muñoz et al., 2017).

Alterations of sleep patterns represent a typical effect of bullying exposure, often persisting over time (Rodríguez‐Muñoz et al., 2020). However, existing research has several limitations. Firstly, most studies have used either cross‐sectional or longitudinal designs with long time lags (ranging between 1 and 2 years). As sleep problems can be an immediate or short‐term response to strain and distress, extended time lags between stressors and outcomes may yield limited conclusions about their relationships. Second, the vast majority of studies rely on self‐reported sleep measures, with scarce information available regarding the impact of bullying on non‐invasive and more objective sleep indicators, such as polysomnography or actigraphy. Among the few exceptions is the study by Pereira et al. (2013), who, using actigraphy‐determined measures of sleep, observed that a construct similar to bullying, namely workplace social exclusion, was associated with poorer sleep quality. In this regard, it can be argued that an individual's subjective perception of sleep may be influenced by their experience of exposure to bullying. Research indicates that targets of bullying often evaluate their environment more negatively, which might lead to reported disruptions in sleep patterns (Rodríguez‐Muñoz et al., 2015). Therefore, considerable knowledge exists regarding the cross‐sectional and longitudinal between‐person level relationship between bullying and sleep. However, there is limited understanding of the within‐person level relationship (Balducci et al., 2024). This distinction is crucial because different levels reveal various aspects of the relationship between workplace bullying and sleep. The within‐person perspective helps us understand immediate psychological responses that affect employees’ daily lives, while the between‐person perspective examines more stable relationships over time. As noted by Bodner and Bliese (2018), between‐person results and mechanisms may not replicate within‐person findings. This study contributes to recent calls in the literature for more research employing a short‐term, within‐person approach and incorporating objective measures of sleep to enhance the methodological quality and rigour of the studies (Nielsen et al., 2020).

Stressful workplace events such as bullying can disrupt sleep, leading to symptoms of insomnia such as difficulty falling asleep, non‐refreshing sleep, and early awakening. Existing evidence suggests that sleep disturbances are practically ubiquitous in situations involving workplace bullying. Across various populations exposed to bullying behaviours, sleep disturbances are frequently reported in both cross‐sectional (Niedhammer et al., 2009; Rodríguez‐Muñoz et al., 2011) and longitudinal studies (Lallukka et al., 2011). Sleep is a complex construct with subjective and objective dimensions. In bullying research, sleep has been predominantly evaluated through self‐reports, which tend to correlate with objective measures but show notable discrepancies. Studies examining sleep through actigraphy‐determined measures have revealed similar effects of stress, yet these results are often less consistent and robust compared to self‐reported sleep (Grafe et al., 2024). Similarly, there is only a moderate correlation between self‐reported and actigraph‐measured sleep duration, with self‐reports typically over‐reporting the duration of sleep (for example, Lauderdale et al., 2008). Additionally, Litwiller et al. (2017) found no significant relationship between subjectively and objectively measured sleep quality in a meta‐analysis. Therefore, the effects of bullying on sleep may be somewhat inflated when relying on subjective measures.

In the present study, we assessed both sleep quality and quantity using actigraphy. In assessing sleep quality, we incorporated actigraphy‐measured parameters such as wake‐after‐sleep onset (WASO: duration of wakefulness occurring after initially falling asleep) and the frequency of awakenings. As for sleep quantity, our primary measure was sleep duration. Both sleep deprivation and sleep fragmentation are two primary types of stress‐induced alterations in sleep patterns (Mezick et al., 2009). Sleep deprivation involves a reduction in sleep duration or outright loss, whereas sleep fragmentation consists of frequent, brief awakenings and heightened arousal levels. Building upon the existing literature, we hypothesised that:

Hypothesis 1 (H1): daily exposure to workplace bullying will be positively associated with (1a) daily sleep fragmentation (i.e., WASO and awakenings) and negatively associated with (1b) daily sleep duration.

Despite strong evidence of the comorbidity between sleep problems and workplace bullying, research on the underlying mechanisms remains limited, particularly in within‐person studies (Nielsen et al., 2020; Rodríguez‐Muñoz et al., 2020). This gap is crucial, as mechanisms identified in between‐person studies may differ from those in within‐person investigations (Balducci et al., 2024). Sleep disturbances can stem from dysfunctional cognitive patterns, maladaptive behaviours, and heightened physiological and mental arousal (Morin, 1993). The hyperarousal model suggests that anxiety triggered by bullying can disrupt sleep by increasing arousal during bedtime, leading to difficulties like prolonged sleep latency and frequent awakenings, thereby suggesting a mediating effect of anxiety on sleep quality (Harvey, 2000). Thus, the abovementioned suggests a mediating effect of anxiety, as proposed in hypothesis 2:

Hypothesis 2 (H2): the within‐individual association between workplace bullying (2a) sleep fragmentation (i.e., WASO and awakenings) and (2b) sleep duration, will be mediated by anxiety.

## 2 METHODS

### 2.1 Participants and procedure

Participants in this study were researchers from a large public university, and data were collected in 2023. Recruitment involves using various social media platforms and advertisements. During face‐to‐face meetings, research assistants instructed participants to complete a general questionnaire before the diary study and were provided with actigraphy devices along with instructions. During the week before the study, participants were required to complete a questionnaire providing general and demographic information. Subsequently, they were tasked with completing a daily questionnaire for 10 consecutive days. To mitigate the confounding influence of weekends on sleep patterns, we specifically selected four consecutive working days from each week for analysis.

This study employed a daily diary research design, resulting in a final sample of 55 participants (*N* = 438 occasions; 65% woman). The mean age of the participants was 29.60 years (standard deviation [SD] = 4.80), with an mean weekly work duration of 41.35 hours (SD = 7.54). Data collection was facilitated using Qualtrics.com, and the research protocol was approved by the local ethics commission. Participants were compensated for their time and incentivised to participate with individualised feedback on their sleep patterns at the end of the study. A post hoc power analysis revealed that our sample size (with power set at 80%) would allow us to detect smaller effects (slopes) than those we observed, indicating that our sample size was more than adequate (Dupont & Plummer Jr, 1998).

### 2.2 Measures

#### 2.2.1 Daily survey

Workplace bullying

Workplace bullying was assessed using the three items derived from the Short‐Negative Acts Questionnaire (Notelaers et al., 2019), which were adapted temporally to capture day‐level experience. Respondents rated each item on a 6‐point Likert scale, ranging from 1 (indicating ‘not true at all’) to 6 (indicating ‘totally true’). Sample items included: ‘Persistent criticism of your work and effort’, and ‘Being ignored or excluded’. The average Cronbach's alpha across waves was 0.72, and the omega reliability coefficient was 0.74.

##### Anxiety

We used two items from the Patient Health Questionnaire (Löwe et al., 2010). Participants indicated how often they ‘felt bothered by’ the following problems during the day: ‘feeling nervous, anxious or on edge’ and ‘not being able to stop or control worrying’. Respondents rated each item on a 6‐point Likert scale, ranging from 1 (indicating ‘not true at all’) to 6 (indicating ‘totally true’). The average Cronbach's alpha across waves was 0.76.

##### Sleep, actigraphy measures

We used wGT3X‐BT actigraphy‐based indicators of sleep outcomes. Actigraphy is an effective way of estimating sleep behaviour, but it does not provide information on sleep stages (Marino et al., 2013). Sleep parameters were derived using integrated algorithms that process movement data collected in 60‐s epochs via ActiLife 6 software (ActiGraph Ltd). Participants were instructed to continuously wear the accelerometer on their non‐dominant wrist for 10 days, with removal permitted only during bathing or showering. Sleep measures obtained from the actigraphy included: sleep duration (total night‐time sleep duration, excluding periods of wakefulness between bedtime and wake time), wake after sleep onset (WASO, minutes awake between sleep onset and final awakening), and the number of awakenings during sleep, all reported in minutes.

#### 2.2.2 General survey

##### Control variables

We adjusted for some control variables in the multilevel regression analyses (age and sex: 1 Male, 2 Woman) because existing literature has consistently shown that the prevalence of sleep problems tends to increase with age and is higher in women compared to men (e.g., Kudielka et al., 2004). Moreover, to control for the cumulative effects of previous days, the variable day was included in the equation.

## 3 RESULTS

### 3.1 Preliminary analyses

Table 1 shows the means, SDs, and correlation for all study variables at the within (below the diagonal) as well as at the between (the person;) level (above the diagonal). At within‐person level, correlations were calculated on the person‐centred variables, to account for the non‐independence of measures. At the between‐person level, correlations related to daily variables were computed based on the average values across measurement occasions. Across both between‐person and within‐person levels, correlations among our key variables exhibited small to moderate magnitudes and were in the expected patterns.

**TABLE 1:** Means, standard deviations, and between‐ and within‐person correlations.

Variable	Mean	SD	Skewness	Kurtosis	1	2	3	4	5	6	7
1) Age	29.61	4.83	3.83	20.1	1						
2) Sex	—	—	—	—	0.17	1					
3) Anxiety	1.76	0.81	1.29	1.23	−0.15	−0.21	1	0.29**	0.18**	0.18**	−0.20**
4) Workplace bullying	1.21	0.57	3.22	10.7	−0.10	−0.10	0	1	0.18**	0.49**	−0.13*
5) WASO	39.58	5.77	0.67	1.57	0.05	−0.24	0.29*	0.11	1	0.26**	0.50**
6) Awakenings	8.01	3.51	2.01	4.98	0.05	−0.20	0.11	0.34**	0.37**	1	0.26**
7) Sleep duration	483.76	83.20	0.87	1.81	0.08	−0.17	−0.18	−0.27*	0.44**	0.37**	1

Abbreviations: SD, standard deviation; WASO, wake‐after‐sleep onset.

*Note*: correlations below the diagonal are person‐level. Correlations above the diagonal are day level (within person).

* *p* < 0.05;

** *p* < 0.01.

### 3.2 Multilevel hypotheses testing

To test our hypotheses, we took into account the nested structure in which daily measurements are nested into individuals. Accordingly, we employed multilevel modelling using R package ‘Multilevel’ (Bliese, 2013) to estimate the multilevel relationship among our variables. Additionally, to test within level mediated relationship we followed the recommendation of Bliese et al. (2018). Specifically, we preliminary tested the null model – (Model 0) to study the variances of our dependent variables (the three objective indicators of sleep quality). Subsequently, in our first model, we introduced our control variables as well as workplace bullying as predictors of anxiety. Subsequently, for each dependent variable, we tested the impact of workplace bullying alone as well as including anxiety. Finally, we used the Sobel test to check the significance of the indirect effect for our H2 (Bliese). Following Bliese et al., 2020, in all the models we controlled for time (day variable in our model) in order to control for unobserved temporal heterogeneity.

Regarding the study of the variance, we computed the intraclass correlation coefficient (ICC)1 for each dependent variable (that informs about the proportion of between level variability over the total amount of variance) and found that a significant amount was related to within level source a variation (necessary to test our hypothesis). The estimated ICC1 were respectively 0.09 for WASO (showing 91% of within level variance approximately), 0.26 for awakenings (showing 74% of within level variance approximately) and 0.26 for sleep duration (showing 74% of within level variance approximately). Those preliminary analysis show that our data were appropriate to test our within level hypotheses.

Regarding the first hypothesis, as shown in Table 2, we found empirical support, showing that workplace bullying is detrimental for sleep quality (respectively *estimate* = 3.77, *p* < 0.01 for WASO; *estimate* = 5.17, *p* < 0.01 for awakening; *estimate* = −36.94, *p* < 0.01 for sleep duration). So, overall, our H1 found empirical support.

**TABLE 2:** Multilevel test of hypothesis.

	Model 1: DV anxiety			Model 2: DV WASO			Model 3: DV WASO			Model 4: DV awakenings			Model 5: DV awakenings		
	Coeff. (SD)	*t*	*p*	Coeff. (SD)	*t*	*p*	Coeff. (SD)	*t*	*p*	Coeff. (SD)	*t*	*p*	Coeff. (SD)	*t*	*p*
Intercept	1.99** (0.51)	3.89	0.00	35.04** (5.61)	6.24	0.00	25.57** (5.69)	5.02	0.00	0.74 (3.24)	0.22	0.82	−0.28 (3.31)	−0.08	0.93
*Control variables*															
Age	−0.01 (0.02)	−0.72	0.47	0.14 (0.16)	0.88	0.37	0.17 (0.15)	1.19	0.26	0.07 (0.09)	0.78	0.44	0.08 (0.09)	0.84	0.39
Sex	−0.05 (0.16)	−0.38	0.73	−2.75 (1.71)	−1.61	0.11	−2.46 (1.69)	−1.51	0.13	−1.19 (0.98)	−1.20	0.22	−1.15 (0.98)	−1.18	0.24
Day	−0.04** (0.01)	−3.08	0.00	0.01 (0.29)	0.04	0.96	0.16 (0.01)	0.56	0.56	0.14 (0.11)	1.31	0.18	0.17 (0.11)	1.51	0.13
*Predictors*															
Anxiety	‐						2.99** (0.87)	3.44	0.00				0.50 (0.38)	1.32	0.19
Workplace bullying	0.31** (0.06)	4.90	0.00	3.77** (1.19)	3.16	0.00	3.05** (0.01)	1.18	0.00	5.17 (0.49)	10.35	0.00	5.01 (0.51)	9.80	0.34
*Indirect effect test parameters*															
Sobel test for indirect effect							0.70 (0.29)		0.00				0.13 (0.09)	0.14	
*Additional information of model estimation*															
AIC	883.02			2745.02			2733.89			2145.56			2145.92		
BIC	910.95			2771.92			2764.62			2172.53			2176.72		
Loglik	−434.51			−1365.51			−1358.94			−1065.78			−1064.96		

	Model 6: Sleep duration			Model 7: Sleep duration		
	Coeff. (SD)	*t*	*p*	Coeff. (SD)	*t*	*p*
Intercept	529.94** (76.51)	6.94	0.00	594.74** (77.15)	7.70	0.00
*Control variables*						
Age	2.15 (2.29)	0.93	0.34	1.83 (2.24)	0.81	0.41
Sex	−35.91 (23.38)	−1.53	0.13	−38.23 (22.90)	−1.66	0.10
Day	−1.04 (2.85)	−0.36	0.71	−2.66 (2.86)	−0.93	0.36
*Predictors*						
Anxiety	‐			−31.60** (9.42)	−3.35	0.00
Workplace bullying	−36.94** (12.50)	−2.95	0.00	−27.88* (12.6°)	−2.21	0.03
*Indirect effect test parameters*						
Sobel test for indirect effect				6.81 (3.02)		0.02
*Additional information of model estimation*						
AIC	4287.38			4271.98		
BIC	4314.18			4302.58		
Loglik	−2136.69			−2127.99		

Abbreviations: AIC, Akaike Information Criterion; BIC, Bayesian Information Criterion; Coeff., coefficient; DV, dependent variable; SD, standard deviation; WASO, wake‐after‐sleep onset.

*Note*: *N* (observations Model 1) = 404; *n* (individuals) = 55 SDs in parentheses.

** *p* < 0.01;

* *p* < 0.05.

Regarding our second hypotheses, we found a positive and significant relationship between workplace bullying and anxiety (*estimate* = 0.31, *p* < 0.01). Additionally, we found a significant indirect effect of workplace bullying and sleep quality through anxiety for two of the three sleep quality indicators, respectively WASO (indirect effect 0.70, *p* < 0.01) and sleep duration (indirect effect 6.81, *p* < 0.01), while the indirect effect for number of awakenings was not significant (indirect effect 0.13, *p* = 0.14). So, overall, we found a partial support for our H2.

## 4 DISCUSSION

To the best of our knowledge, this is the first study to examine how daily exposure to workplace bullying is related to objective sleep fragmentation disturbances (i.e., shorter duration and increased fragmentation). Our initial findings indicate that bullying significantly disrupts sleep in the short term. Sleep fragmentation, as indicated by waking after sleep onset and the frequency of awakenings during sleep, is positively associated with daily exposure to bullying behaviours. Similarly, bullying is linked to reductions in sleep duration on a daily basis. These findings are consistent with prior research, which, utilising subjective sleep scales, identified workplace bullying as a significant contributor to short‐ and long‐term sleep‐related complaints (e.g., Nielsen et al., 2020), difficulty initiating sleep (Niedhammer et al., 2009), awakening problems (Hansen et al., 2014), and WASO (Rodríguez‐Muñoz, 2020).

The high comorbidity between sleep problems and workplace bullying across various contexts and populations may suggest that sleep disturbances could potentially act as indicators of a transdiagnostic factor in stress‐related situations (Cox & Olatunji, 2020). Disruptions in sleep may reflect imbalances in the arousal system, a key factor in mental health. A study found that insomnia symptoms persisted even after the bullying ceased (Rodríguez‐Muñoz et al., 2020), with sleep and stress responses sharing biological pathways (Åkerstedt et al., 2002). This study also examines whether daily anxiety mediates the relationship between bullying and sleep. Results suggest anxiety mediates the impact on sleep duration and WASO, but not on the frequency of awakenings, highlighting a focus on sleep quantity rather than frequency. These findings partially support the hyperarousal model of sleep (Riemann et al., 2010).

The study's robustness includes using actigraphy to obtain non‐intrusive measurements of participants’ sleep over multiple days. Despite the strengths of the design employed, our study had some limitations. First, the generalisability of our findings is possibly restricted due to the use of a convenience sample sourced exclusively from a single occupational group at one university. As our participants had higher education levels than the general Spanish workforce, they are not fully representative. Hence, future studies should aim to replicate the results of this research in more diverse populations. Another limitation of our study may be due to potential seasonal biases. The data collection occurred at two separate time points during the year, specifically in May and November, which potentially introduced seasonal variations that could have influenced the results. Nevertheless, we conducted an initial analysis controlling for this factor, and no significant differences were observed.

Regarding the practical implications, addressing the primary prevention of bullying in organisations is crucial. However, there is also a clear need to address sleep‐related issues. It appears that workplace bullying not only directly impacts sleep quality but also exacerbates anxiety, further disrupting sleep patterns. Therefore, interventions aimed at mitigating the impact of bullying once it occurs should also include strategies to improve sleep hygiene, such as psychoeducational programmes on sleep hygiene, and address sleep disturbances among affected individuals. This implies that organisations and support systems should not overlook the importance of sleep in the context of the workplace, as it constitutes a crucial aspect of employees’ overall well‐being and resilience (Barling et al., 2016). Moreover, organisations must not only implement policies and procedures to prevent and address bullying but also provide resources and support mechanisms to help employees cope with its psychological effects. This may involve investing in mental health resources, offering training programmes that promote a culture of respect and inclusion, and providing access to interventions aimed at improving sleep quality. For example, cognitive behavioural therapy, relaxation exercises, and stress management strategies can empower individuals to regulate arousal levels and promote better sleep hygiene. By addressing both bullying and its psychological effects, organisations can create healthier, more supportive environments.

## AUTHOR CONTRIBUTIONS

**Alfredo Rodríguez‐Muñoz:** Conceptualization; investigation; funding acquisition; writing – original draft; writing – review and editing; methodology; supervision; project administration. **Mirko Antino:** Methodology; conceptualization; investigation; funding acquisition; writing – review and editing; formal analysis; software. **Alejandro Díaz‐Guerra:** Data curation; software; formal analysis; methodology; writing – review and editing; project administration. **Ana Isabel Sanz‐Vergel:** Conceptualization; investigation; writing – review and editing. **Arnold B. Bakker:** Writing – review and editing; investigation; conceptualization.

## CONFLICT OF INTEREST STATEMENT

All authors declare that they have no conflicts of interest.

## Data Availability

The data that support the findings of this study are openly available in Zenodo Data repository at https://zenodo.org/, reference number https://doi.org/10.5281/zenodo.10996662.
